# SW-UNet: a U-Net fusing sliding window transformer block with CNN for segmentation of lung nodules

**DOI:** 10.3389/fmed.2023.1273441

**Published:** 2023-09-28

**Authors:** Jiajun Ma, Gang Yuan, Chenhua Guo, Xiaoming Gang, Minting Zheng

**Affiliations:** ^1^Shenhua Hollysys Information Technology Co., Ltd., Beijing, China; ^2^The First Affiliated Hospital of Dalian Medical University, Dalian, China; ^3^School of Software, North University of China, Taiyuan, China; ^4^Anshan Municipal Central Hospital, Anshan, China

**Keywords:** medical image segmentation, attention mechanism, Vision Transformer, lung nodule, tumor

## Abstract

Medical images are information carriers that visually reflect and record the anatomical structure of the human body, and play an important role in clinical diagnosis, teaching and research, etc. Modern medicine has become increasingly inseparable from the intelligent processing of medical images. In recent years, there have been more and more attempts to apply deep learning theory to medical image segmentation tasks, and it is imperative to explore a simple and efficient deep learning algorithm for medical image segmentation. In this paper, we investigate the segmentation of lung nodule images. We address the above-mentioned problems of medical image segmentation algorithms and conduct research on medical image fusion algorithms based on a hybrid channel-space attention mechanism and medical image segmentation algorithms with a hybrid architecture of Convolutional Neural Networks (CNN) and Visual Transformer. To the problem that medical image segmentation algorithms are difficult to capture long-range feature dependencies, this paper proposes a medical image segmentation model SW-UNet based on a hybrid CNN and Vision Transformer (ViT) framework. Self-attention mechanism and sliding window design of Visual Transformer are used to capture global feature associations and break the perceptual field limitation of convolutional operations due to inductive bias. At the same time, a widened self-attentive vector is used to streamline the number of modules and compress the model size so as to fit the characteristics of a small amount of medical data, which makes the model easy to be overfitted. Experiments on the LUNA16 lung nodule image dataset validate the algorithm and show that the proposed network can achieve efficient medical image segmentation on a lightweight scale. In addition, to validate the migratability of the model, we performed additional validation on other tumor datasets with desirable results. Our research addresses the crucial need for improved medical image segmentation algorithms. By introducing the SW-UNet model, which combines CNN and ViT, we successfully capture long-range feature dependencies and break the perceptual field limitations of traditional convolutional operations. This approach not only enhances the efficiency of medical image segmentation but also maintains model scalability and adaptability to small medical datasets. The positive outcomes on various tumor datasets emphasize the potential migratability and broad applicability of our proposed model in the field of medical image analysis.

## 1. Introduction

The segmentation of medical images plays a crucial role in the analysis of medical imaging, involving the separation and labeling of distinct regions within such images (1). This technology holds significant importance in various medical applications, such as tumor diagnosis (2). Medical image segmentation techniques assist physicians in precisely identifying tumor regions, measuring tumor dimensions and shape, and offering valuable insights for devising treatment strategies (3, 4). In recent times, the fusion of computer-aided detection (CAD) and deep learning has emerged as a prominent area of research in medical image segmentation (5). This is due to the following advantages: medical image segmentation tasks can extract specific regions for quantitative analysis and calculation, providing more objective and accurate results for diagnosis and treatment, which in turn improves patient treatment results; medical image segmentation algorithms can provide accurate references for doctors' diagnosis and treatment decisions, reducing their workload and shortening the time for diagnosis and treatment; medical image segmentation topics are computer medical image segmentation is a hot topic in the field of computer vision technology, and the study of medical image segmentation algorithms can promote the development and application of artificial intelligence technology in the field of medicine and provide reference and reference for the application in other fields. Machine learning uses algorithmic models that allow computers to learn the context of visual data on their own (6).

The current mainstream medical image segmentation models are mainly segmentation models, and most of the existing models adopt the structure of CNN as the main framework, thanks to two inductive biases: local correlation and weight sharing, which are assumptions that can help the network to learn and generalize effectively in the case of insufficient training data or high noise (7). Although CNN-based approaches perform well in tasks in computer vision, the bias assumption of convolutional operations also limits the performance of the model in learning remote dependencies to local perceptual fields (8), thus losing the possibility of capturing long-range feature associations and not being flexible enough to adapt to image inputs of different sizes, shapes, and textures, leading to information loss and model instability (9–12).

Vaswani et al. (13) proposed a new convolution-independent model, Transformer, in which the traditional CNN and RNN are discarded and the entire network structure is composed entirely of Attention mechanisms. More precisely, Transformer consists of and only consists of Self-Attention and Feed Forward Neural Network. A trainable neural network based on Transformer can be built by stacking Transformers, and the authors' experiments were conducted by building Encoder-Decoder with 6 layers each of encoder and decoder, totaling 12 layers, and achieved a new high BLEU worth in machine translation. The use of attentional mechanisms and multiscale analysis methods are widely used in the field of medical analysis as well as in other fields (14–16). Dosovitskiy et al. (17) proposed ViT that divides the input image into multiple patches, and then projects each patch into a fixed-length vector to be fed into the Transformer, with the subsequent encoder operations identical to those in the original Transformer. Liu et al. (18) proposed a Swin Transformer with a hierarchical design that includes sliding window operations, in response to the problem that Transformer's computation based on global self-attention leads to a large amount of computation. The sliding window operation includes a non-overlapping local window, and an overlapping cross-window. Limiting the attention computation to a single window introduces the localization of the CNN convolution operation on the one hand, and saves computation on the other (19).

Inspired by the attention mechanism in natural language processing (13, 17, 18), existing studies use the Transformer, a non-local neural network, to overcome this limitation, which can model remote dependencies in sequence-to-sequence tasks, capturing relationships between arbitrary positions in a sequence (20). The Transformer structure is proposed based only on the self-attention mechanism, completely eliminating the convolutional structure, and is powerful in modeling global context is powerful, and several studies have shown that Transformer-based frameworks also achieve state-of-the-art performance on a variety of computer vision tasks (21). However, the self-attentiveness in Transformer requires large computation and memory consumption when dealing with long sequences, and the sparse nature of medical image data makes the model prone to overfitting during the training session, which hinders the application of Transformer in medical image segmentation tasks, which has been tried and tested in the field of natural image processing (22, 23). To reduce the number of computational parameters, we refer to the approach in Swin Transformer, which uses two layers of attention structures with a hierarchical design, including a non-overlapping local window, and an overlapping cross-window (18).

Our main contributions are:

- We propose a medical image segmentation network SW-UNet based on a hybrid CNN-ViT architecture;
- We design a Transformer module with a sliding window design to overcome the lack of interaction between different regions of conventional convolutional operations and reduce the number of stacked modules by widening the self-attentive vector dimension to the effect of reducing the model parameters is achieved;
- Validating the effect of our model on lung nodule dataset LUNA16 and other tumor datasets, the model yields consistent improvements over many baselines.

## 2. Method

**Figure 1:**
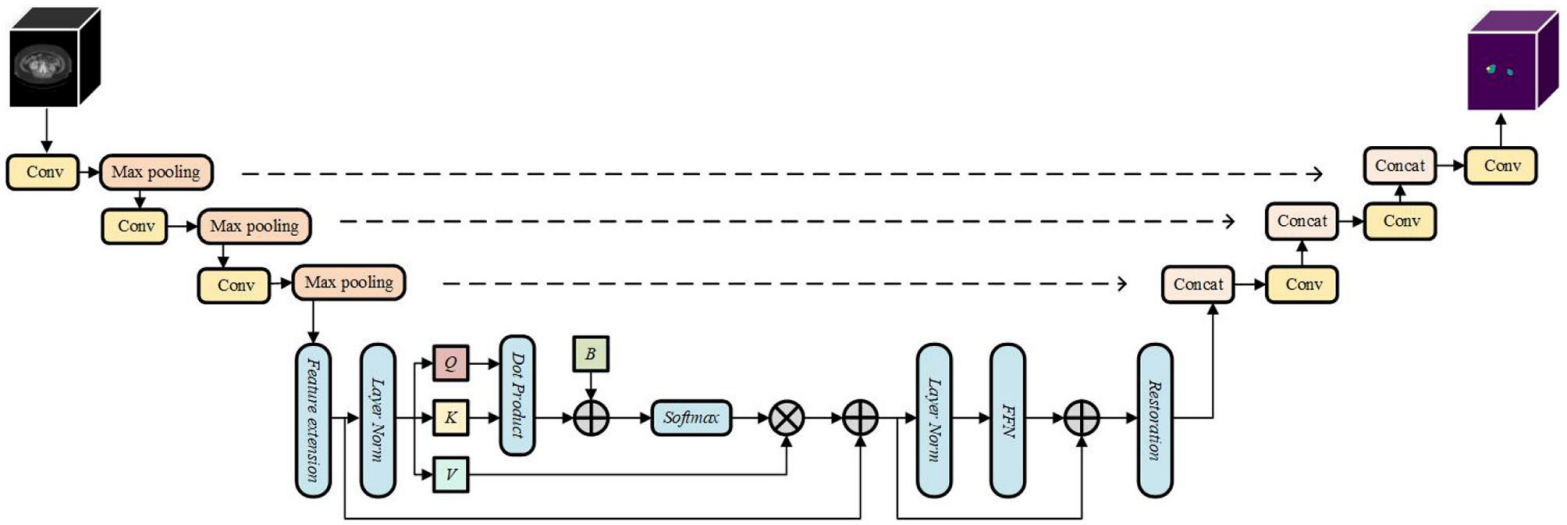
The structure of the model.

In this paper, we design a CNN-ViT based medical image hybrid segmentation network SW-UNet, taking into account the strengths of both. First, the CNN is able to rapidly compress the number of input feature image pixels in the downsampling phase as a way to reduce the computational cost of the whole model, which allows the model to be trained and inferred faster. Second, ViT achieves long-range sequence modeling through a self-attentive mechanism, which can tap the degree of association between arbitrary pixel points. This global perception capability can help the model better understand the overall structure of the image, rather than just segmenting local regions. For some images with strong global structure features, ViT can outperform the traditional CNN model. the design of SW-UNet combines the advantages of CNN and ViT, making full use of the features of both models, thus enabling the model to better adapt to different medical image datasets. The structure of the model is shown in Figure 1.

### 2.1. Encoders and decoders

The architecture of the SW-UNet model is based on the encoder-decoder structure of U-Net. Initially, the input image *F*∈*R*^*C*×*H*×*W*^ undergoes a 3 × 3 convolution operation, followed by three consecutive downsampling steps using convolution with a stride of 2. This downsampling process aims to reduce the size of the feature map before inputting it into the final ViT. Our input is a single-channel 1 × 128 × 128 CT image map of the lungs, and both the encoder and decoder have a three-layer structure, with each layer consisting of two consecutive convolutions; the pooling layer in the encoder reduces the image height and width by half, and the transposed convolution in the decoder doubles the image size.

By employing this encoding step prior to inputting into ViT, the SW-UNet model efficiently extracts local image features, allowing for effective utilization of computational resources in the early stages of the model. The resulting feature map is then fed into the Sliding Window Transformer Block (SWTB) for further operations.

On the decoder side, a similar structure to the encoder is employed. Each layer is upsampled using two successive 3 × 3 deconvolutions, and the encoder features are fused with the decoder features at each layer through skip connections. This fusion enables the SW-UNet model to capture finer spatial details in the segmentation process.

### 2.2. Sliding window transformer block

Since the computational complexity of the ViT structure is proportional to the square of the input sequence length, it is impractical to directly tile the input image into a sequence as the input to the model. Therefore, the feature map needs to be segmented into fixed-size chunks as the sequence input before it is passed into ViT. For the feature map output at the encoding side, we design a feature expansion module to make the dimensionality of the feature map meet the input requirements of ViT. First, the number of channels of the feature map is expanded from 64 by the convolution operation, and the two dimensions of H and W are combined into the same dimension to finally obtain the input sequence $z_{0}\in R^{d\times N}$ of SWTB, where $N=\frac{H}{4}\times \frac{W}{4}$ is the number of elements corresponding to the incoming sequence in ViT.

Specifically, the computation of attention is accomplished in two stages: in the first stage, the computation of attention is performed inside the regular non-overlapping window, and in the second stage, the computation of attention is performed in the new window obtained by shifting the first layer of the window to the right by a distance of half of the window's width, i.e., the sliding window operation. The difference in complexity between the self-attention computation without the sliding window operation and the self-attention computation with the sliding window operation is shown in Equations (1) and (2), respectively, where *H*, *W*, and *C* represent the height, width, and number of channels of the feature map, respectively, and M represents the length of the rectangular window edge in terms of feature points as a preset unit. For the experimental setup in this chapter, the computation of the single self-attention structure is reduced from 800 million reduced to 270 million, which effectively relieves the computational pressure of the model as a whole.


(1) $$\begin{array}{l} \text{Ω}\left(W-MSA\right)=4HWC^{2}+2H^{2}W^{2}C \end{array}$$

(2) $$\begin{array}{l} \text{Ω}\left(SW-MSA\right)=4HWC^{2}+2M^{2}HWC \end{array}$$

**Figure 2:**
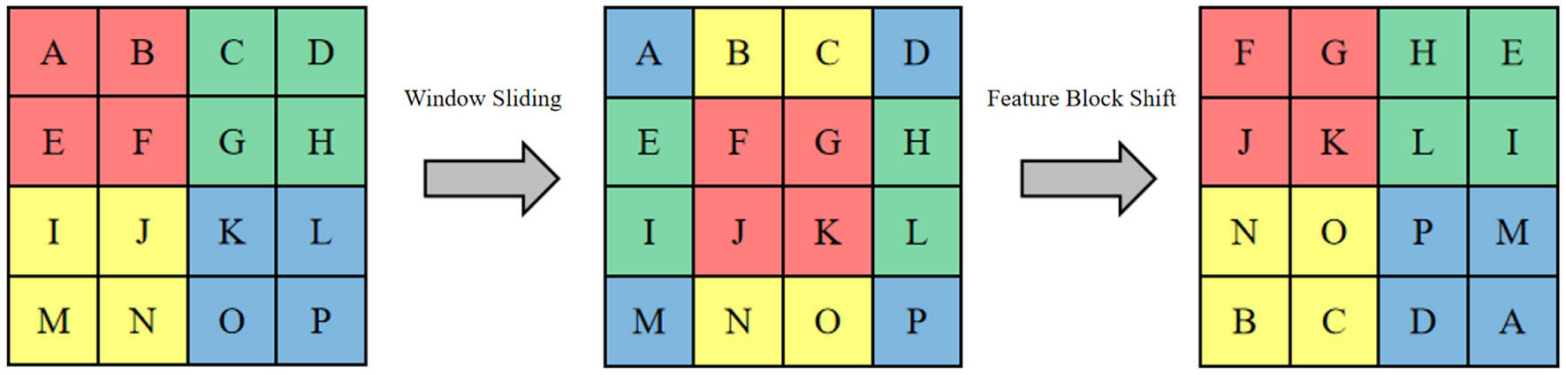
The shifting window operation and feature shift operation.

In order to alleviate the problem of large computational effort of traditional ViT in computing global attention of images, we design SWTB with sliding window operation and layer design to replace the global multi-headed self-attention mechanism, to save computational effort by limiting the attention computation to a window of fixed size, so that the computational complexity grows linearly instead of squarely with the image size, and to allow correlation between windows so as to achieve The first layer performs the multi-headed self-attentive computation inside a regular non-overlapping window, and the second layer performs the multi-headed self-attentive computation in a new window obtained by shifting the first window down to the right by a distance of half the window width, which is also known as the sliding window operation. Specifically, the spatial dimension of the input sequence *z*_0_ is *N* = 32 × 32. To facilitate the edge operation, we set the window size to *M* = 4 × 4 and the window is panned down to the right by 2 pixel points at a time. This is shown in Figure 2, where the letters represent different feature sequences and the colors represent the range of receptive fields for different windows.

**Figure 3:**
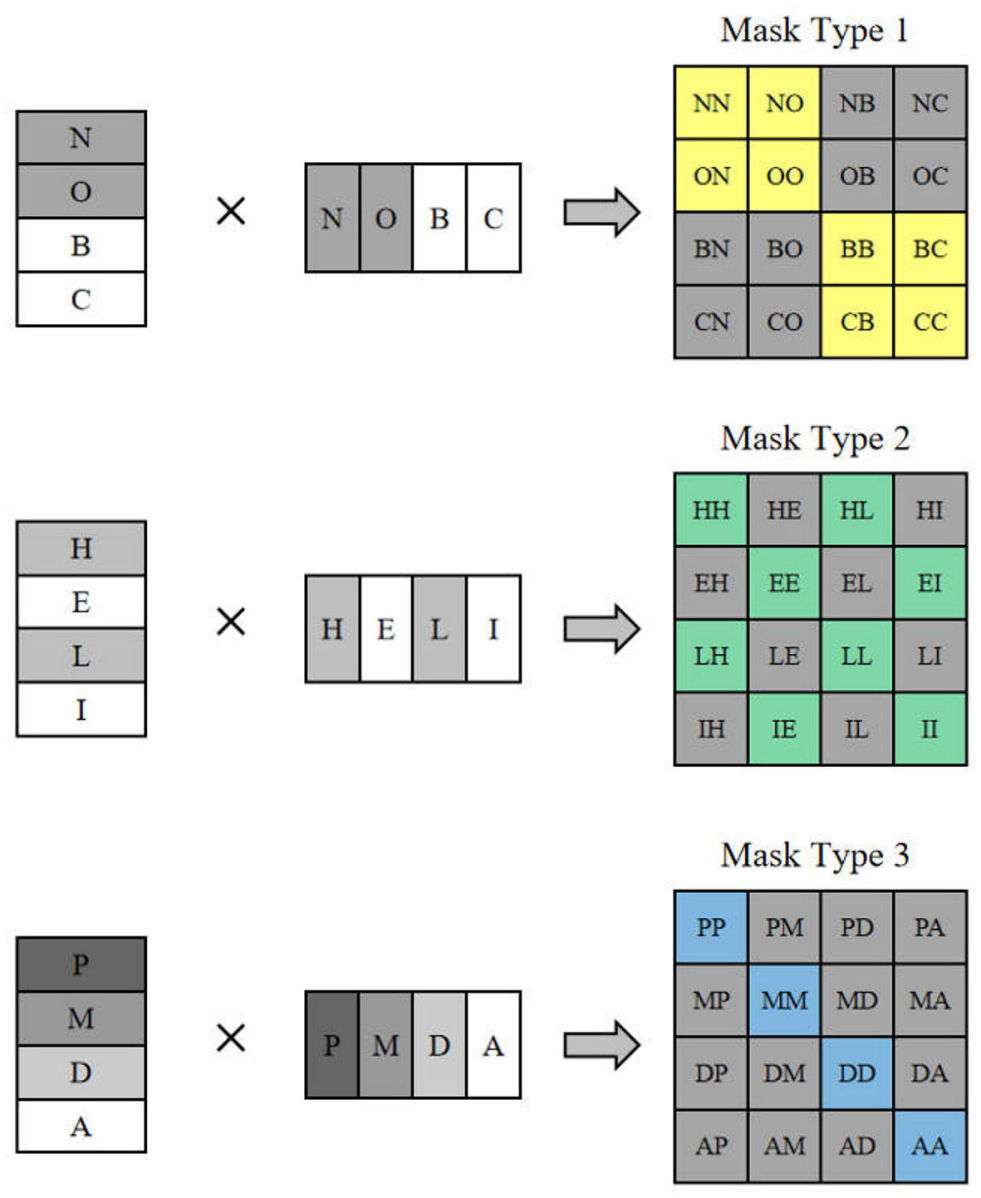
The mask type corresponding to feature shift operation.

Since only half a window distance is shifted, a feature map of half a window width will appear at the edge of the whole feature map, if this part is directly discarded it will result in the loss of feature information, and if this part is retained it will introduce additional computation, both of which are not very desirable. For this reason, this section proposes a sliding window operation with cyclic shifting, where the extra part is added to other small windows to make them complete windows, as shown in Figure 3. This cyclic shifting brings a new problem: for some windows, some of their feature blocks are moved from other positions, and these feature blocks are subject to self-attention computation with originally non-adjacent feature blocks, so that the values obtained from this computation lack practical significance, and are only operated in this way in order to keep the number of windows the same as the original. In order to block out the interference of these pseudo-values, three different types of masks are designed here to be added with the results of the self-attention calculation.

Many previous investigations have utilized multiple sets of Transformer modules with numerous self-attentive mechanisms computed sequentially to enhance their efficacy. While this approach can effectively enhance the model's performance, it also introduces a substantial number of parameters, which we believe have more adverse effects than positive advantages for medical image segmentation tasks. To mitigate the proliferation of parameters associated with stacking Transformer modules, we reduce the depth of the SWTB while increasing its width. Specifically, we augment the width of ViT using the Feature Expansion module before input, and we expand the hidden layer dimension of Q and K to *d*_*m*_ = *Ed* by increasing the rate E while keeping the dimension of V unchanged. Within the SWTB, the self-attention computation for each window follows the equations shown in Equations (3) and (4). In these equations, $W_{q}\in R^{d\times d_{m}}$, $W_{k}\in R^{d\times d_{m}}$, and $W_{v}\in R^{d\times d}$ represent the parameter matrices, and the features $x_{i}\in R^{M^{2}\times d}$ of each window are multiplied with these parameter matrices to yield three attention vectors: $Q\in R^{M^{2}\times d_{m}}$, $K\in R^{M^{2}\times d_{m}}$, and *V*∈*R*^*M*^^2^×*d*. To incorporate positional information in the self-attention within the window, the calculation integrates a learnable relative positional deviation *B*∈*R*^*M*^^2^×*M*^2^. Here, we set the number of groups for the multi-headed self-attention mechanism to 4. The features obtained from the final computation are concatenated, and the information is further consolidated through an additional global self-attention layer.


(3) $$\begin{array}{l} \left[Q;K;V\right]=\left[W_{q};W_{k};W_{v}\right]\cdot x_{i} \end{array}$$

(4) $$\begin{array}{l} Attention\left(Q,K,V\right)=Softmax\left(\frac{QK^{T}}{\sqrt{d}}+B\right)V \end{array}$$

Equations (5), (6), (7), and (8) depict the equations pertaining to the entirety of the ViT segment. Here, the notation LN denotes layer normalization, and FFN represents the output of the window multi-headed self-attention mechanism, as well as serving as the input for the sliding window multi-headed self-attention mechanism. The sliding window multi-headed self-attention mechanism part generates the output for further processing.


(5) $$\begin{array}{l} z_{0}^{\prime }=W-MSA\left(LN\left(z_{0}\right)\right)+z_{0} \end{array}$$

(6) $$\begin{array}{l} z_{1}=FFN\left(LN\left(z_{0}^{\prime }\right)\right)+z_{0}^{\prime } \end{array}$$

(7) $$\begin{array}{l} z_{1}^{\prime }=SW-MSA\left(LN\left(z_{1}\right)\right)+z_{1} \end{array}$$

(8) $$\begin{array}{l} z_{2}=FFN\left(LN\left(z_{1}^{\prime }\right)\right)+z_{1}^{\prime } \end{array}$$

After the feature map has gone through SWTB, the model has learned enough image information. In order to achieve a skip connection and keep the feature map in the same number of layers with the same size in the encoder, two consecutive convolution operations are used here to reshape its dimension to size $d\times \frac{H}{4}\times \frac{W}{4}$. After the above operations, the feature map has the same shape as the output *F* at the encoder side.

## 3. Experiments

### 3.1. Datasets

We use the segmentation results of the algorithm on the LUNA16 lung nodule dataset (24) to evaluate the performance of the SW-UNet model proposed in this section. To verify the general applicability of the model to medical images, we conduct experiments on two other tumor datasets, the LiTS 2017 (25) liver tumor dataset and the KiTS 2019 (26) kidney tumor dataset. The details of the three datasets are described below.

#### 3.1.1. Lung nodule dataset LUNA16

The LUNA16 dataset is a subset of the largest public lung nodule dataset, LIDC-IDRI, whose main purpose is to perform automatic detection and segmentation of lung cancer. 888 CT scans of the lung are included in the LUNA16 dataset, each containing 1–4 lesions, for a total of 1,186 lesions. The LUNA16 annotations are presented in terms of nodule location (x, y, and z-axis coordinates), and the original image size is 512 × 512.

#### 3.1.2. Liver tumor segmentation dataset LiTS 2017, kidney tumor segmentation challenge dataset KiTS 2019

The LiTS 2017 dataset is provided by the Liver Tumor Segmentation (LiTS) Challenge 2017, which has 200 3D CT scan images, including 130 images for model training and validation and 70 images for objective model evaluation. Similar to LiTS 2017, KiTS 2019 is also provided by the Kidney Tumor Segmentation (KiTS) Challenge 2019. The dataset has a total of 300 3D CT scans containing 210 images for model training and validation and 90 images for objective model evaluation, with image sizes of 512 × 512 pixels for both datasets.

### 3.2. Model evaluation metrics

In this paper, the evaluation of the model is mainly done using the Dice coefficient and the Hausdorff 95 (H95) distance, etc. The Dice coefficient is calculated as shown in Equation (9). The Dice coefficient measures the similarity between the predicted and true results, and its value ranges from 0 to 1. The closer the value is to 1, the higher the similarity between the predicted and true results, and the better the performance of the model. On the contrary, the closer the value is to 0, the lower the similarity between predicted and true results, and the worse the performance of the model.


(9) $$\begin{array}{l} Dice=\frac{2TP}{2TP+FP+FN} \end{array}$$

The Hausdorff distance is calculated as shown in Equation 10, which measures the similarity between two sets, and is calculated by calculating the minimum value of the distance from each point in set *X* to set *Y*, and then taking the maximum value of these minimum values as the Hausdorff distance, which is commonly used in medical image segmentation to measure the difference between the segmentation result of the model and the real segmentation result. The smaller the H95 value, the smaller the difference between the segmentation result of the model and the real segmentation result, and the better the segmentation performance of the model.


(10) $$\begin{array}{l} Hausdorff=max\left\{d\left(X,Y\right),d\left(Y,X\right)\right\} \end{array}$$

### 3.3. Data pre-processing and experimental parameter setting

In order to make the data better suited to the network structure proposed in this work, a preprocessing operation is required on the data. On the three datasets, the range of pixel values saved in their raw data varies due to different criteria. In order to have the same distribution of grayscale values for each image in the training set, pixel value normalization of the input data is necessary. Specifically, for the background and content-containing pixels in medical images, MinMax normalization is applied in each image, calculated as shown in Equation (11). After performing the above operation, the pixel values are all distributed between 0 and 1. For the LUNA 16 dataset, because of its special annotation form, we first generate the corresponding masks indicating the nodal locations using the annotations and sliced them to fit our 2D network model. Since it contains large non-nodal regions, we crop the image according to the nodal locations and selected an image of 128 × 128 size including all parts of the nodes. Similarly, in order to keep the image size of the incoming model consistent, we uniformly adjust the images of the LiTS 2017 dataset and KiTS 2019 dataset to 128 × 128 by cropping or resampling means to fit the network parameters.


(11) $$\begin{array}{l} x_{i}^{\prime }=\frac{x_{i}-min\left(x_{i}\right)}{max\left(x_{i}\right)-min\left(x_{i}\right)} \end{array}$$

We use the deep learning library Pytorch to build the model and to train it. In the training session, we use an initial number of 16 convolutional kernels and set each batch to 48. We use Adam as the optimizer with a learning rate of 0.00001 and end the training with convergence after 100 rounds with no decrease in the loss value on the validation set. For each dataset, we divide the data that can participate in training into a training set and a validation set, where the training set accounts for 80%, the validation set accounts for 10%, and the remaining 10% is used as a test set to evaluate the model. Our loss function continues to use the combined loss of Dice loss and Focal loss, where the weight α of Dice loss is set to 0.8 and the weight β of Focal loss is set to 0.2.


(12) $$\begin{array}{l} \begin{array}{l} L_{Dice}=1-\frac{2\sum_{i}^{N}y_{i}y_{i}^{\prime }+\varepsilon }{{\sum_{i}^{N}y_{i}}_{i}+\sum_{i}^{N}y_{i}^{\prime }+\varepsilon } \end{array} \end{array}$$

(13) $$\begin{array}{l} L_{Focal}=\sum_{i}^{N}\left(-y_{i}{\left(1-y_{i}^{\prime }\right)}^{\gamma }logy_{i}^{\prime }-(1-y_{i})y_{i}^{\prime \gamma }log(1-y_{i}^{\prime })\right) \end{array}$$

(14) $$\begin{array}{l} L_{Seg}=\alpha L_{Dice}+\beta L_{Focal} \end{array}$$

### 3.4. Results

To verify the performance of SW-UNet, we compare its segmentation results with those of TransUNet (27), TransAttnet (28), and TransBTS (29), and Tables 1–3 lists their performance when running on LUNA16, LiTS 2017, and KiTS 2019. It can be seen that SW-UNet achieves optimal performance in all two metrics, Dice coefficient and H95 distance. In the Dice coefficient score, SW-UNet has a small lead of about 1-5 percentage points on all three datasets, and it can be said that SW-UNet is more accurate and clearer for the segmentation effect of the internal structure of the model. In the evaluation index of H95 distance, SW-UNet achieves 7.41, 9.13, and 8.26 scores, respectively, which also achieves the best, which indicates that SW-UNet is also very accurate in dividing the prominent part of the edge region.

**Table 1:** Segmentation results of different models on LUNA 16.

**Method**	**Accuracy**	**Dice**	**Sensitivity**	**Specificity**	**H95**
TransUNet	0.98	0.79	0.80	0.98	13.46
TransAttUnet	**0.99**	0.81	**0.82**	0.98	9.12
TransBTS	**0.99**	0.83	0.81	**0.99**	7.55
SW-UNet	**0.99**	**0.84**	**0.82**	**0.99**	**7.41**

The bolded ones are the best results.

**Table 2:** Segmentation results of different models on LiTS 2017.

**Method**	**Accuracy**	**Dice**	**Sensitivity**	**Specificity**	**H95**
TransUNet	0.97	0.58	0.60	0.98	13.83
TransAttUnet	0.98	0.63	0.62	0.98	11.65
TransBTS	0.98	0.62	0.62	**0.99**	9.76
SW-UNet	**0.99**	**0.66**	**0.65**	**0.99**	**9.13**

The bolded ones are the best results.

**Table 3:** Segmentation results of different models on KiTS 2019.

**Method**	**Accuracy**	**Dice**	**Sensitivity**	**Specificity**	**H95**
TransUNet	0.98	0.77	0.75	0.98	11.34
TransAttUnet	0.98	0.80	0.78	0.98	10.19
TransBTS	**0.99**	0.80	0.77	0.98	8.85
SW-UNet	**0.99**	**0.82**	**0.81**	**0.99**	**8.26**

The bolded ones are the best results.

To make the model suitable for medical image segmentation tasks, we reduce the number of model parameters as much as possible so that it can be trained efficiently without overfitting problems even with only a small amount of data. As shown in Table 4, we count the number of parameters for several models with Transformer structure. Among them, the number of parameters of SW-UNet is only 32M, which is 63% lower than the 86M parameters of ViT-Base, 70% lower than the 105M parameters of TransUNet, and 25% lower than the 43M parameters of TransBTS. It can be said that SW-UNet is a structure specialized for medical image segmentation tasks, and for those diseases with distinct geographical features, each hospital has the ability to label a small dataset by itself for training without acquiring huge amount of data.

**Table 4:** Comparison of different models.

**Model**	**Vision Transformer-base**	**TransUNet**	**TransBTS**	**SW-UNet**
Parameters	86M	105M	43M	**32M**
FLOPs	**33.03G**	1205.76G	333.09G	51.3G

Bolded values are the ones that achieve the best performance in the network performance comparison.

### 3.5. Visualization analysis

**Figure 4:**
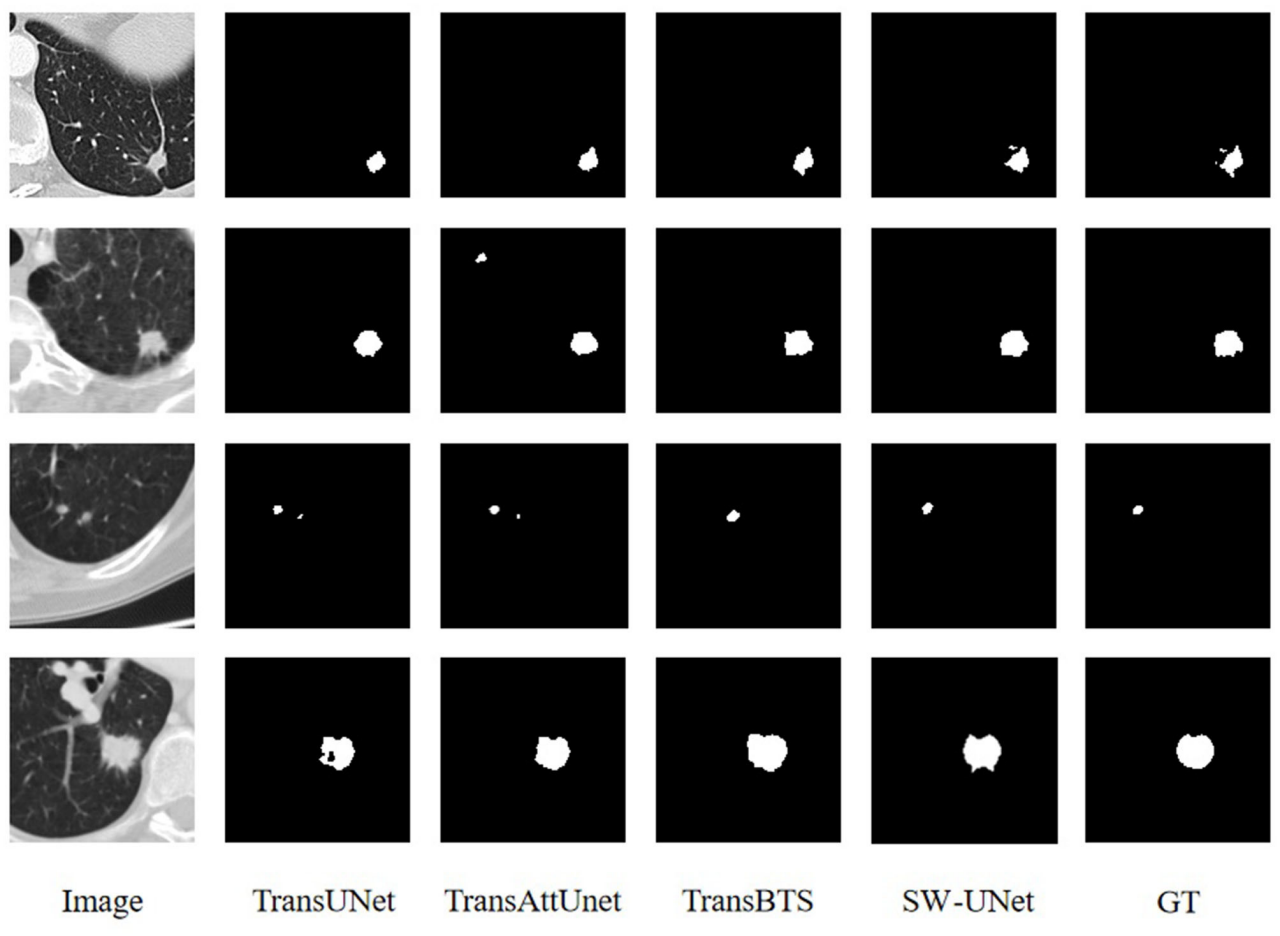
Visualization of segmentation results for LUNA16 dataset.

To visually evaluate the model performance, we randomly select four sets of segmentation results of TransUNet, TransAttUnet, TransBTS and SW-UNet on three datasets, LUNA 16, LiTS 2017, and KiTS 2019, as a display. As shown in Figure 4, the black part of the LUNA 16 segmentation results is the background and the white part is the segmentation results. It can be seen that part of the segmentation results of TransUNet has deviations in positioning, segmenting parts that originally do not belong to the label, and there are also errors in the division and positioning of the interior of the segmented region. TransAttUnet is not accurate enough in portraying the shape of the segmented region, and also has the problem of incorrectly identifying the background as the segmented region. TransBTS has a fair performance in segmenting the smoother trend of the The segmentation performance of TransBTS is fair at the edges, but it cannot detect protrusions and small fragmented areas. In contrast, SW-UNet is more accurate in shape description, and its localization performance and anti-interference ability are also more outstanding.

**Figure 5:**
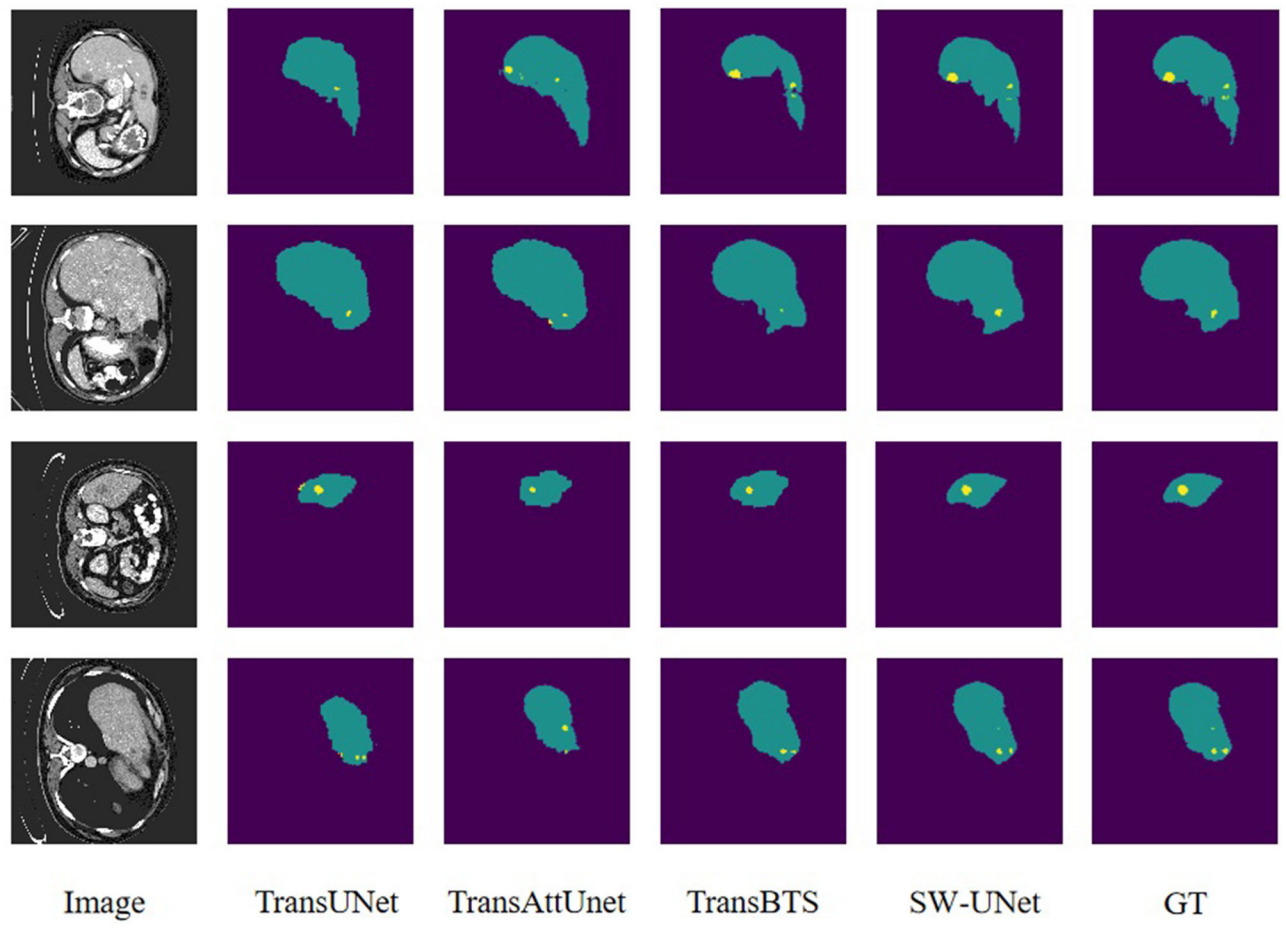
Visualization of segmentation results for LiTS 2017 dataset.

**Figure 6:**
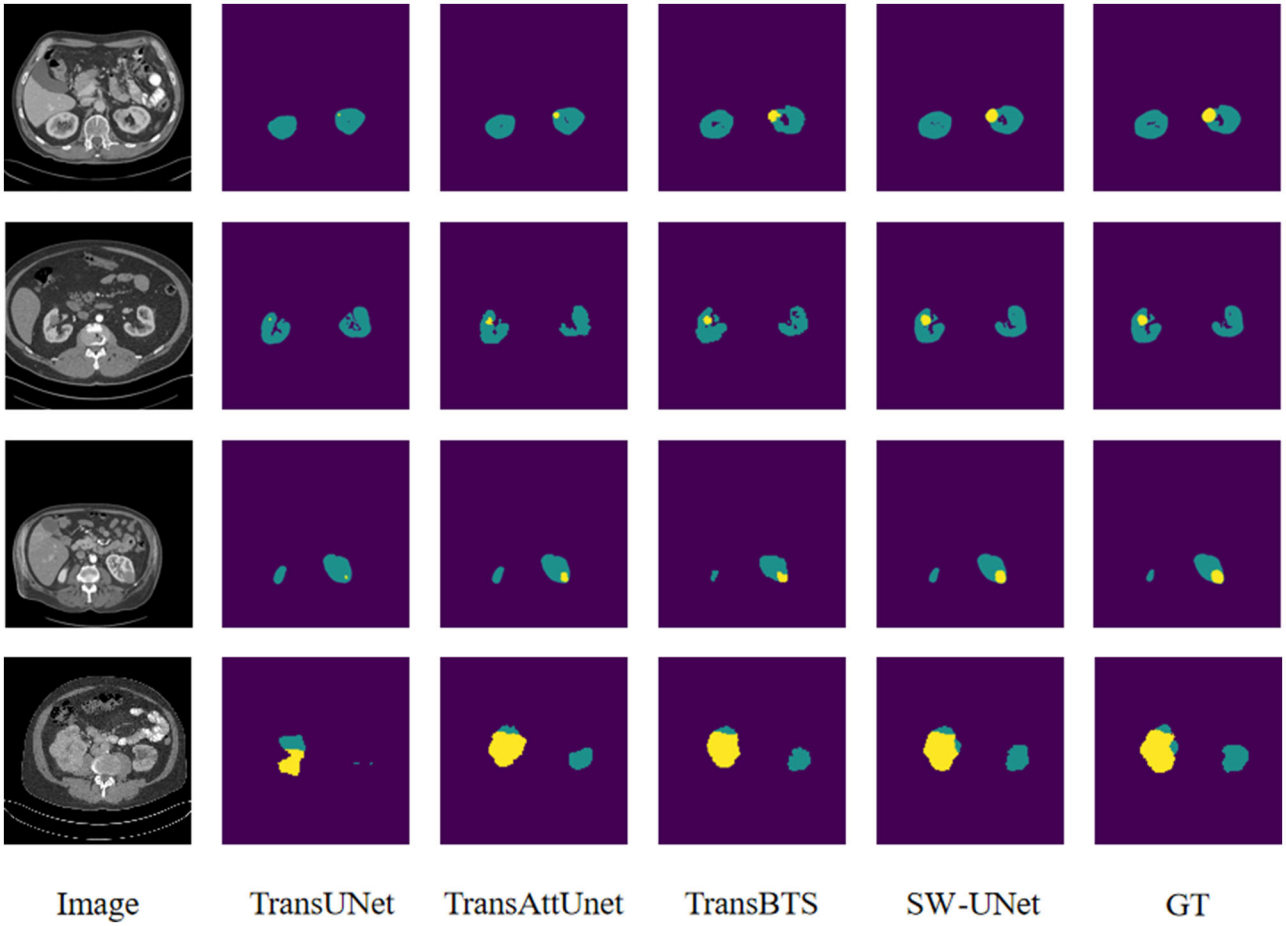
Visualization of segmentation results for KiTS 2019 dataset.

The images of the LiTS 2017 and KiTS 2019 datasets have many similarities, mainly in that the tumor regions in both datasets are very small compared to the organ parts. Therefore, it is not too difficult for the model to segment out the organ part, while it is more difficult to precisely locate the tumor region. The purple part of the segmentation results of both datasets is the background, the green part is the organ segmentation results, and the yellow part is the tumor segmentation results. From Figures 5, 6, we can see that TransUNet can only segment the organ part in many samples, but cannot perceive the tumor region, especially in those LiTS samples with low contrast of the original image. TransBTS is closer to the real value in organ segmentation, but the recognition performance of tumor region is slightly inferior to that of SW-UNet. The segmentation results of both organs and tumor regions are very accurate.

Taken together, our proposed SW-UNet model is more accurate for the internal details of the segmented region, and the boundary alignment of the segmentation is clearer, which can better capture the minute structures and changes in the images and improve the accuracy of the segmentation. Due to the complexity of medical image structure and artifact interference, general models may have incorrect segmentation. The segmentation results on three important human organs, lung, kidney, and liver, prove that the SW-UNet model has strong generalization and good robustness, and can be migrated to other data sets for training and specialization to segmentation models for specific organs or specific disease types, thus helping This will help doctors to better understand and diagnose patients' conditions.

### 3.6. Ablation experiments

To verify the improvement of SWTB on model segmentation performance, we design ablation experiments to test the impact of SWTB on overcoming the bias assumption of the CNN architecture, and the control group replaced the sliding window module in SWTB with a non-sliding module. As can be seen in Table 5, the models with the sliding window mechanism added are generally higher in Dice coefficient scores by more than 5 percentage points compared to the models without the sliding window mechanism on the three different datasets, which indicates that the association of perceptual fields established between different windows can bring positive effects on the model segmentation performance. The Transformer module, which is the core operation to establish the association between different perceptual fields, is no different from the convolution operation in the CNN framework, as both of them compute the features in part of the feature map, except that the CNN part can be optimized in terms of computational efficiency to better adapt to the training and inference needs of large-scale datasets. It can also be seen from Table 5 that the model with the sliding window mechanism has smaller values of the H95 distance metric compared to the model without the sliding window mechanism, indicating that the contours of the segmented regions are also better portrayed and can adapt to the edges of the regions with different scale size variations.

**Table 5:** The ablation study results of sliding window operation.

Dataset	**Dice**		**H95**	

	**No SW**	**With SW**	**No SW**	**With SW**
LUNA16	0.79	0.84	21.38	7.41
LiTS 2017	0.59	0.66	12.87	9.13
KiTS 2019	0.75	0.82	15.42	8.26

In addition, we also experimentally investigate the optimal solution for the number of CNN downsampling times to find the optimal placement of the encoder by varying the number of times the encoder is downsampled at the input SWTB, set to downsample once, twice, and three times, respectively. As can be seen in Table 6, the data after downsampling twice achieve the highest scores in the Dice coefficient evaluation index, which indicates that the combination of high-level semantic and low-level features after downsampling twice gives the best segmentation effect to the model for the input image. In addition, the data after three downsampling are better and closer to the results after two downsampling than after one downsampling, and even better than the model after two downsampling on the H95 evaluation metric for the LiTS 2017 dataset, suggesting that the computation on high-level semantics is more valuable compared to doing the computation on global self-attentive mechanism on low-level features. However, we believe that the conclusion may have some limitations due to the fact that the size of the input image is fixed at 128 × 128, which might have different standard answers in different segmentation tasks if it is properly adjusted in the data preprocessing stage.

**Table 6:** The ablation study results of CNN downsampling frequency.

Dataset	**Dice**			**H95**		

	**1**	**2**	**3**	**1**	**2**	**3**
LUNA16	0.79	**0.84**	0.81	11.03	**7.41**	8.97
LiTS 2017	0.58	**0.66**	0.65	14.62	9.13	**8.78**
KiTS 2019	0.76	**0.82**	0.80	15.18	**8.26**	9.44

Bolded values are those that achieved the best performance in the down sampling count comparison.

## 4. Conclusion

This paper presents the design ideas and experimental results of a lung nodule image segmentation model based on a hybrid architecture of CNN and ViT. First, the universal problems of segmentation networks based on CNN architecture are analyzed, and the negative effects caused by their inductive bias are analyzed and solutions are given. Secondly, the detailed design process and implementation method of SW-UNet, the sliding window Transformer module, the medical image segmentation model proposed in this chapter are given. Finally, the effectiveness of the SW-UNet model is verified by experiments on lung nodule dataset LUNA 16, and the general applicability of the model for medical image segmentation is confirmed on two other datasets.

## Data availability statement

The original contributions presented in the study are included in the article/supplementary material, further inquiries can be directed to the corresponding author.

## Author contributions

JM: Writing—original draft, Writing—review editing. GY: Writing—review and editing, Validation. CG: Writing—original draft, Writing—review and editing. XG: Writing—review and editing, Investigation, Resources. MZ: Investigation, Validation, Writing—original draft, Writing—review and editing.

## References

[1] Gao W Li X Wang Y Cai Y. Medical image segmentation algorithm for three-dimensional multimodal using deep reinforcement learning and big data analytics. Front Public Health. (2022) 10:879639. 10.3389/fpubh.2022.879639 35462800 PMC9024167

[2] Zhao L Lin R Liu Z Yuan H. Predicting the likelihood of patients developing sepsis based on compound ensemble learning. In: 2022 IEEE International Conference on Bioinformatics and Biomedicine (BIBM). Las Vegas, NV (2022). p. 3235–41.

[3] He K Lian C Zhang B Zhang X Cao X Nie D. HF-UNet: learning hierarchically inter-task relevance in multi-task U-Net for accurate prostate segmentation in CT images. IEEE Trans Med Imaging. (2021) 40:2118–28. 10.1109/TMI.2021.3072956 33848243

[4] Jin Q Meng Z Sun C Cui H Su R. RA-UNet: a hybrid deep attention-aware network to extract liver and tumor in CT scans. Front Bioeng Biotechnol. (2020) 8:605132. 10.3389/fbioe.2020.605132 33425871 PMC7785874

[5] Wang R Lei T Cui R Zhang B Meng H Nandi AK. Medical image segmentation using deep learning: a survey. IET Image Process. (2022) 16:1243–67. 10.1049/ipr2.12419

[6] Zhao L Huang P Chen T Fu C Hu Q Zhang Y. Multi-sentence complementarily generation for text-to-image synthesis. IEEE Trans Multimedia. (2023) 1–10. 10.1109/TMM.2023.3297769

[7] Peng J Wang Y. Medical image segmentation with limited supervision: a review of deep network models. arXiv preprint arXiv:2103.00429 (2021). 10.48550/arXiv.2103.00429

[8] Wu M Qian Y Liao X Wang Q Heng PA. Hepatic vessel segmentation based on 3D swin-transformer with inductive biased multi-head self-attention. BMC Med Imaging. (2021) 23:91. 10.1186/s12880-023-01045-y 37422639 PMC10329304

[9] Dai D Li Y Wang Y Bao H Wang G. Rethinking the image feature biases exhibited by deep convolutional neural network models in image recognition. CAAI Trans Intell Technol. (2022) 7:721–31. 10.1049/cit2.12097

[10] Chai J Zeng H Li A Ngai EWT. Deep learning in computer vision: a critical review of emerging techniques and application scenarios. Mach Learn Appl. (2021) 6:100134. 10.1016/j.mlwa.2021.100134

[11] Guo F Ng M Goubran M Petersen SE Piechnik SK Neubauer S. Improving cardiac MRI convolutional neural network segmentation on small training datasets and dataset shift: a continuous kernel cut approach. Med Image Anal. (2020) 61:101636. 10.1016/j.media.2020.101636 31972427

[12] Liu L Wang Y Chang J Zhang P Liang G Zhang H. LLRHNet: multiple lesions segmentation using local-long range features. Front Neuroinform. (2022) 16:859973. 10.3389/fninf.2022.859973 35600503 PMC9119082

[13] Vaswani A Shazeer N Parmar N Uszkoreit J Jones L Gomez AN. Attention is all you need. In: Proceedings of the 31st International Conference on Neural Information Processing Systems. NIPS'17. Red Hook, NY, USA: Curran Associates Inc. (2017). p. 6000–10.

[14] Zou L Huang Z Yu X Zheng J Liu A Lei M. Automatic detection of congestive heart failure based on multiscale residual UNet++: from centralized learning to federated learning. IEEE Trans Instrument Meas. (2023) 72:1–13. 10.1109/TIM.2022.3227955

[15] Zou L Qiao J Yu X Chen X Lei M. Intelligent proximate analysis of coal based on near infrared spectroscopy and multi-output deep learning. IEEE Trans Artif Intell. (2023) 1–13. 10.1109/TAI.2023.3296714

[16] Zhao L Yang T Zhang J Chen Z Yang Y Wang ZJ. Co-learning non-negative correlated and uncorrelated features for multi-view data. IEEE Trans Neural Netw Learn Syst. (2021) 32:1486–96. 10.1109/TNNLS.2020.2984810 32356763

[17] Dosovitskiy A Beyer L Kolesnikov A Weissenborn D Zhai X Unterthiner T. An image is worth 16x16 words: transformers for image recognition at scale. arxiv preprint arXiv:2010.11929. 10.48550/arXiv.2010.11929

[18] Liu Z Lin Y Cao Y Hu H Wei Y Zhang Z. Swin transformer: hierarchical vision transformer using shifted windows. arxiv preprint arXiv:2103.14030. 10.48550/arXiv.2103.14030

[19] Sun W Chen J Yan L Lin J Pang Y Zhang G. COVID-19 CT image segmentation method based on swin transformer. Front Physiol. (2022) 13:981463. 10.3389/fphys.2022.981463 36072854 PMC9441795

[20] Yan S Wang C Chen W Lyu J. Swin transformer-based GAN for multi-modal medical image translation. Front Oncol. (2022) 12:942511. 10.3389/fonc.2022.942511 36003791 PMC9395186

[21] Valanarasu JMJ Oza P Hacihaliloglu I Patel VM. Medical transformer: hierarchical vision transformer using shifted windows. In:de Bruijne M Cattin PC Cotin S Padoy N Speidel S Zheng Y et al., editors. Medical Image Computing and Computer Assisted Intervention – MICCAI 2021. Cham: Springer International Publishing (2021). p. 36–46.

[22] Ding Y Jia M Miao Q Cao Y. A novel time–frequency Transformer based on self–attention mechanism and its application in fault diagnosis of rolling bearings. Mech Syst Signal Process. (2022) 168:108616. 10.1016/j.ymssp.2021.108616

[23] Huang L Zhu E Chen L Wang Z Chai S Zhang B. A transformer-based generative adversarial network for brain tumor segmentation. Front Neurosci. (2022) 16:1054948. 10.3389/fnins.2022.1054948 36532274 PMC9750177

[24] Shukla VVK Tanmisha M Aluru R Nagisetti B Tumuluru P. Lung nodule detection through CT scan images and DNN models. In: 2021 6th International Conference on Inventive Computation Technologies (ICICT). Coimbatore (2021). p. 962–7.

[25] Bilic P Christ P Li HB Vorontsov E Ben-Cohen A Kaissis G. The liver tumor segmentation benchmark (LiTS). Med Image Anal. (2023) 84:102680. 10.1016/j.media.2022.102680 36481607 PMC10631490

[26] Heller N Sathianathen NJ Kalapara AA Walczak E Moore K Kaluzniak H. The KiTS19 challenge data: 300 kidney tumor cases with clinical context, CT semantic segmentations, and surgical outcomes. arXiv preprint arXiv:1904.00445. 10.48550/arXiv.1904.00445

[27] Chen J Lu Y Yu Q Luo X Adeli E Wang Y. TransUNet: transformers make strong encoders for medical image segmentation. arXiv preprint arXiv:2102.04306. 10.48550/arXiv.2102.0430637109505

[28] Chen B Liu Y Zhang Z Lu G Zhang D. TransAttUnet: multi-level attention-guided U-Net with transformer for medical image segmentation. arXiv preprint arXiv:2107.05274. 10.48550/arXiv.2107.05274

[29] Wang W Chen C Ding M Yu H Zha S Li J. TransBTS: multimodal brain tumor segmentation using transformer. In:de Bruijne M Cattin PC Cotin S Padoy N Speidel S Zheng Y et al., editors. Medical Image Computing and Computer Assisted Intervention – MICCAI 2021. Cham: Springer International Publishing (2021). p. 109–19.

